# A Rapid Method for Refolding Cell Surface Receptors and Ligands

**DOI:** 10.1038/srep26482

**Published:** 2016-05-24

**Authors:** Lu Zhai, Ling Wu, Feng Li, Robert S. Burnham, Juan C. Pizarro, Bin Xu

**Affiliations:** 1 Department of Biochemistry, Virginia Polytechnic Institute & State University, Blacksburg, VA 24061, USA; 2 Interdepartmental Microbiology Graduate Program, Virginia Polytechnic Institute & State University, Blacksburg, VA 24061, USA; 3 Department of Tropical Medicine, Tulane University, New Orleans, LA 70112, USA; 4 Center for Drug Discovery, Virginia Polytechnic Institute & State University, Blacksburg, VA 24061, USA.

## Abstract

Production of membrane-associated cell surface receptors and their ligands is often a cumbersome, expensive, and time-consuming process that limits detailed structural and functional characterization of this important class of proteins. Here we report a rapid method for refolding inclusion-body-based, recombinant cell surface receptors and ligands in one day, a speed equivalent to that of soluble protein production. This method efficiently couples modular on-column immobilized metal ion affinity purification and solid-phase protein refolding. We demonstrated the general utility of this method for producing multiple functionally active immunoreceptors, ligands, and viral decoys, including challenging cell surface proteins that cannot be produced using typical dialysis- or dilution-based refolding approaches.

Cell surface immunoreceptors, ligands and viral decoys (receptors and ligands) belong to a major class of membrane receptors that play vital roles in a variety of biological processes. These include molecular recognition, viral infection, and immune defense. The successful expression and purification of these host and microbial surface proteins in active forms are critical for biochemical, structural, and functional studies of receptor-ligand molecular recognition in the context of host-pathogen interactions. Among the variety of challenges associated with the study of this class of molecules, obtaining purified functional receptor and ligand proteins is often a long and labor-intensive process.

Because of the limitations in obtaining targeted cell surface molecules from native tissues and cells expressing the proteins, recombinant forms of receptor and ligand proteins are often the preferred choice for investigations. Among the heterologous systems used to express target proteins (integral membrane proteins excluded), bacteria hosts represent the most economical system in terms of associated costs, speed, and ease of use. However, cell surface proteins partition to the inclusion body (IB) fractions in majority cases when overexpressed in *E. coli*. This makes inclusion body-based receptor/ligand refolding often a required step to obtain functionally active cell surface proteins (for reviews on general methods in inclusion body-based protein refolding, see references^1^,^2^,^3^). Multiple cases of refolding-based receptor production have been reported^4^,^5^,^6^,^7^,^8^,^9^. Traditional chaotrope-based refolding techniques, however, require purified inclusion bodies of the target proteins of interest, which in turn require extensive washing steps (traditional solid-phase or centrifugation-based) that can take multiple days (Fig. 1A and Supplemental Figure S1). Typical refolding approaches involve either step-wise gradient dilution to dialyze away high concentrations of chaotrope agents such as urea or drop-wise dilution of denatured inclusion bodies directly into a refolding buffer. The entire process of refolding for cell surface proteins can span from one to over two weeks (Fig. 1A and Supplemental Figure S1)^4^,^10^, yet the success rates of these refolding approaches are usually low^11^. Lengthy turn-around times, labor-intensive processes, and low chances of successful refolding make these refolding approaches daunting. Therefore, investigators need to expend significant amounts of time to find optimal conditions in a trial-and-error fashion. During these lengthy processes, the amount of chemical and biochemical reagents consumed to aid the refolding process (such as large quantities of the chaotrope reagents urea and guanidine hydrochloride (GuHCl), protein aggregation inhibiting-reagents such as arginine, reducing and oxidizing reagents, etc.) is considerable. Despite all the technical challenges, these approaches are routinely used to generate many cell surface receptor and ligand proteins with intact native structures and active functions^3^,^4^,^5^,^6^,^7^,^8^,^9^. On-column refolding of proteins (including receptor fragments or domains) using immobilized metal ion affinity chromatography (IMAC) has been reported in several studies^6^,^7^,^8^. These studies were typically using highly purified IBs before on-column refolding and each refolding was usually optimized for a single target.

One approach to cope with technical challenges in protein refolding is to apply high throughput screening. Developments of such refolding screenings have been reported, based on analytical detection of reverse phase-fast protein liquid chromatography (rp-FPLC) or differential scanning fluorimetry (DSF)^12^,^13^. These studies focused on expanding the capability to accommodate large numbers of proteins. For example: establishing a 96-well-plate format so that refolding conditions may be optimized rapidly.

We sought to develop a rapid and general method for refolding cell surface receptors and ligands that will fill a much needed technology gap for an important class of biomacromolecules in structural biology, functional genomics, and chemical biology applications. Built upon works from literature and our systematic efforts to refold a variety of cell surface immuno- and viral receptors and ligands, we report here a rapid and less expensive method that successfully refolded membrane-associated receptors and ligands with different classes of structures and functions.

## Results

**Figure 1:**
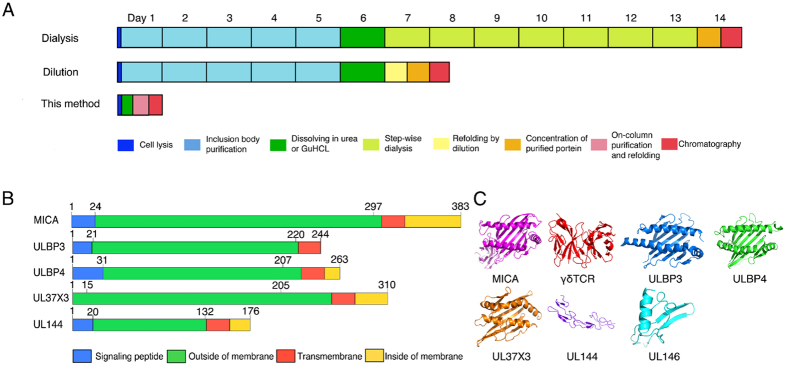
Schematic representations of time required for refolding in different methods, illustration of targeted cell surface proteins sequence domains, and protein structures and models. (**A**) Schematic comparison of different refolding methods with respect to time consumed. Major steps are represented by the following color-coded boxes: dark blue, cell lysis; light blue, inclusion body purification (including IB purity check using SDS-PAGE); green, dissolving in urea or GuHCl; lemon, step-wise dialysis; yellow, refolding by dilution; orange, concentration of refolded proteins; pink, on-column purification and refolding; red, chromatography. (**B**) Predicted sequence boundaries for the targeted cell surface proteins with regard to the transmembrane segment. Extracellular domain of each membrane protein was chosen for expression and refolding (green domains). (**C**) Predicted structures of the targeted proteins of interest. Protein Data Bank (PDB) code 1HYR, 3OMZ, and 1KCG were used to display the structures of MICA, γδ TCR, and ULBP3 respectively. Structural models for the remaining targets were generated using programs Phyre2 or I-Tasser^29^,^30^.

To solve the problems associated with regular refolding methods, we sought to dramatically enhance the efficiency of the steps used to purify the inclusion bodies. To bypass the slow and labor-intensive washing steps of inclusion bodies in their solid-state form, we chose to directly solubilize the inclusion bodies in a chaotrope solution after cell disruption. We then coupled affinity purification of chaotrope-dissolved proteins and refolding steps with IMAC via a Ni-NTA column to streamline the entire operation. Seamless connection between on-column purification and on-column refolding is possible in part due to that Ni-NTA or similar resins used for IMAC purification can tolerate the chaotropic buffers and mild redox conditions. An additional advantage of these procedures is that the elution volumes of refolded targets can be easily controlled (i.e., using smaller volume to elute) to avoid an extra concentration step for the final preparative protein liquid chromatography (Fig. 1A and Supplemental Figure S1).

We have chosen multiple cell surface immunoreceptors and viral decoy receptors as targets to test our method and to evaluate its efficacy across a variety of classes of receptor and ligand proteins (Fig. 1B,C). The selection was also designed to include several different scenarios on targets: receptors/ligands that can be refolded by the traditional dialysis approach, ones that cannot be refolded, and ones that are unknown. These targets include MHC class I like immunoreceptors and related viral mimics (MICA^14^, ULBP3^5^, ULBP4^15^, exon 3 of HCMV UL37 (UL37 × 3)^16^), T-cell receptor (γδ TCR)^9^, TNF receptor mimic (UL144)^17^, and chemokine mimic (UL146)^18^. To our knowledge, production or refolding of several predicted viral surface receptors (ULBP4, UL37 × 3, UL144, and UL146) have not been reported, while refolding of MICA, ULBP3, and γδ TCR immuno-ligands and immunoreceptors have been reported^4^,^5^,^9^. In our expression construct design, we used pET22b or pET15b vectors and excluded signal peptides and transmembrane segments since the ectodomains of most immunoreceptors or viral receptors are responsible for their molecular recognition functions (Fig. 1B). For γδ TCR, we joined the Vγ and Vδ domains with a flexible link to make a single chain construct mimicking scFv^9^. One of these seven proteins, UL146, is a viral chemokine mimic and it does not contain a transmembrane segment^18^. All constructs included a his_6_-tag in their amino-termini or carboxyl-termini to facilitate affinity chromatographic purification (see Supplemental Table S1).

Our method consists the following four steps: cell lysis, dissolution of raw inclusion bodies in chaotropic reagents, on-column purification and refolding, and size exclusion chromatography (Fig. 1A and Supplemental Figure S1). When compared to classical “dialysis” or “dilution” approaches, this method avoids two lengthy steps: inclusion body washing and step-wise dialysis of dissolved inclusion bodies. These two steps consume 70–90% of the time required for a standard refolding process. Using our approach, a refolding trial can be completed in a single day compared to typically more than one week required for the “dilution” (if the IB washing steps are counted) or the “dialysis” methods for many receptor proteins (Fig. 1A and Supplemental Figure S1)^19^,^20^. Without counting the lengthy inclusion body washing steps, this method is still significantly more efficient in terms of time and effort.

We choose 3 M and 1.5 M urea buffers as key intermediate steps between the fully denatured state (8 M urea) and fully folded state (0 M urea). These steps are intended to stabilize intermediate folding or unfolded states of the proteins, which may in turn facilitate the refolding reactions to be more productive^21^.

**Figure 2:**
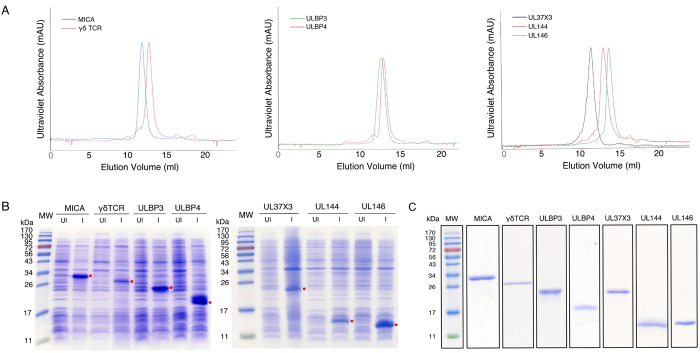
Recombinant protein expression, rapid refolding, and purification. (**A**) FPLC size exclusion purification of all seven immunoreceptors and viral cell surface proteins. (**B**) Whole cell lysates were analyzed using 15% SDS-PAGE gels. In both panels, the molecular weight standards (MW) are shown in the first lanes. Un-induced (UI) and induced (I) samples were loaded in neighboring wells for each target. The proteins of interest were highlighted with red dots on the right. (**C**) SDS-PAGE of purified cell surface receptors and ligands.

Disulfide bond formation contributes significantly to the stability of the native conformation of many proteins and is a key parameter for efficient refolding reactions^19^. In eukaryotes, disulfide bond formation occurs during protein folding through a complex process that takes place primarily in the endoplasmic reticulum and that is catalyzed by the protein disulfide isomerase enzyme in the presence of millimolar concentrations of oxidized and reduced glutathione^22^. Disulfide bonds can be stably maintained only in an oxidizing environment. Because the cytoplasm of the *E. coli* host is a reducing environment, proteins that require disulfide bonds cannot be made efficiently in typical competent *E. coli* strains. In certain instances, secondary structure formation may be so favorable that the correct cysteine residues spontaneously bind and only weakly oxidizing conditions are required. However, if this is not the case, stronger oxidizing conditions may be required. We have tested a number of reducing or reducing/oxidizing reagents in our system. Three redox pairs, cysteamine/cystamine, cysteine/cystine, and reduced glutathione (GSH)/oxidized glutathione (GSSG) successfully refolded target proteins (data for sample target ULBP3 is shown in Supplemental Figure S2), but not reducing agents β-mercaptoethanol (βME), dithiothreitol (DTT), tris(2-carboxyethyl)phosphine (TCEP) (data not shown; high concentrations of βME and DTT may also reduce and inactivate Ni-NTA resins)^23^. Based on relative yields of correctly folded proteins, we selected the redox pair of GSH and GSSG that creates the necessary oxidizing potential to make and break disulfide bonds in folding intermediates, thereby allowing the optimal native conformation to be reached. The choice of GSH/GSSG also reflects the fact that this redox pair occurs *in vivo* during protein folding^22^. With GSH:GSSG redox pair, we further tested the refolding of ULBP3 by varying their ratio in the refolding buffers. We found that different redox ratios chosen had no significant difference on ULBP3 yields (Supplemental Figure S2). A GSH:GSSG ratio of 10:1 (at a concentration of 1–5 mM GSH) was selected because this condition was effective for refolding all the proteins in this work (Fig. 2A,C).

The yields of refolded and purified receptor proteins using this method are overall comparable with those using regular dialysis or dilution approaches with the exception of UL37 exon 3. We used yields from literature or from our own work with regular dialysis and dilution approaches as reference values for comparison. Detailed yields and quality of purified target proteins are shown in Table 1, Fig. 2A–C. We were able to refold exon 3 of UL37 from HCMV, a MHC class I-like viral mimic, using this method, but not with regular dialysis or dilution methods despite multiple trials. To our knowledge, this is the first reported production of purified HCMV protein UL37 exon 3. We speculate that the success of this refolding by our rapid method is likely due to the efficiency of on-column purification and refolding procedures and the favorable environment created. The UL37 case highlights the potential of our method as it provides an alternative for proteins with low refolding efficiencies. A similar case has been reported where the L2 domain of the epidermal growth factor receptor (EGFR) could be refolded only through the on-column approach, but not dilution or stepwise dialysis strategies^24^. Using two target proteins, ULBP3 and UL146, as examples, we quantified protein recovery/loss in each step of our procedures in comparison with those in the traditional “dialysis” approach. Amounts of target proteins recovered from each step were quantified and the results were shown in Supplemental Table S2.

**Figure 3:**
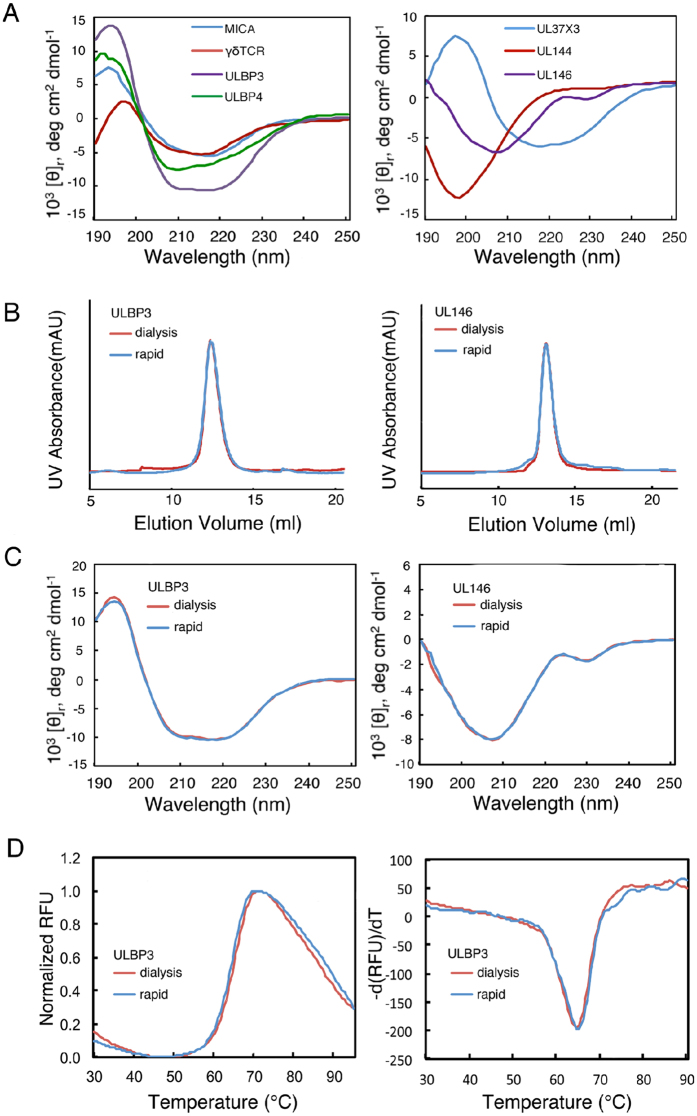
Circular dichroism and differential scanning fluorimetry analyses of the refolded proteins. (**A**) CD profiles of target proteins. Left panel: MICA, blue; γδ TCR, red; ULBP3, purple; ULBP4, green; Right panel: exon 3 of UL37; blue; UL144, red; UL146, purple. The spectra were acquired using 5–20 μM protein concentrations in 10 mM potassium phosphate buffer, pH 7.0. (**B**) Comparison of size exclusion chromatography FPLC profiles of selected proteins ULBP3 and UL146. (**C**) Comparison of circular dichroism secondary structure profiles of selected proteins ULBP3 and UL146. (**D**) Comparison of thermo-unfolding curves of ULBP3 refolded from our rapid refolding and traditional dialysis methods. Left panel shows the normalized relative fluorescence response and right panel shows the first derivative of the thermo-melting curve (-*d*(RFU)/*d*t)^26^,^32^.

To verify if the purified target molecules folded correctly, we used circular dichroism (CD) to evaluate the secondary structures of all seven cell surface proteins (Fig. 3A). As expected, spectra of MICA, ULBP3, ULBP4, UL37 × 3 displayed α-helices-dominant secondary structures (negative peaks at wavelengths of 208 nm, 222 nm, or both). The spectrum of UL146 was consistent with a typical chemokine with mixed α-helix and β-strand components. γδ TCR displayed a spectrum of a typical β-strand rich protein (negative peak at 215 nm) and viral receptor mimic UL144, a predicted TNF receptor homolog, displayed an extended random coil secondary structure. Its secondary structure as measured by circular dichroism was consistent with our predicted three-dimensional structure (Figs 1C and 3A). We hypothesize that the transmembrane segment of UL144 may be required to form a folded, oligomeric structure similar to that of the human TNF receptor^25^. To further verify that the proteins were correctly refolded, we compared the FPLC profiles and circular dichroism spectra of proteins refolded and purified by our method with those of the traditional dialysis method as controls. Using ULBP3 and UL146 as two examples, proteins refolded by either method elute at identical retention volume (Fig. 3B). ULBP3 proteins, refolded by two different methods, have superimposable secondary structures (Fig. 3C). The same are true for UL146 proteins (Fig. 3C). Further we used the differential scanning fluorimetry (DSF) method to compare protein thermostability using ULBP3 as an example. Themo-melting profiles of ULBP3 refolded with either method were superimposable too, taking experimental noises into account (Fig. 3D). The UL146 protein does not have significant signals in DSF analysis, likely due to its small size (15 kD) and low percentage of structured elements in the protein to bind fluorescent dye (data not shown)^26^.

**Figure 4:**
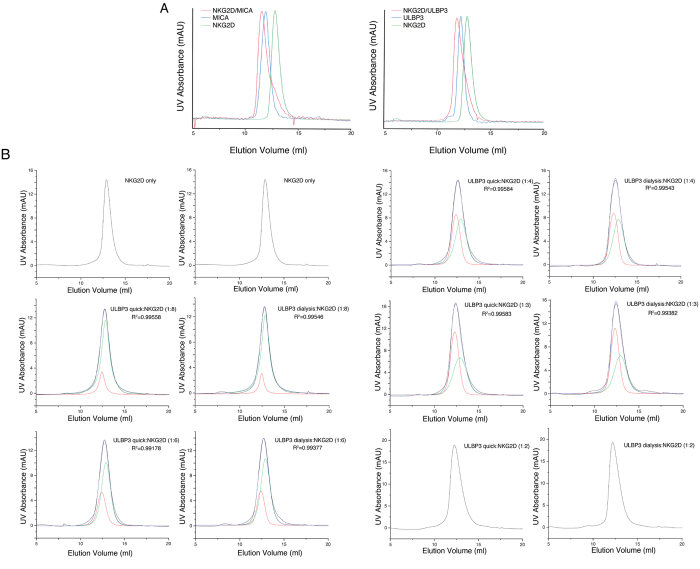
Functional binding characterizations of selected refolded proteins. (**A**) Binding Size exclusion chromatography FPLC profiles of cell surface protein alone (MICA or ULBP3, blue traces), receptor only (NKG2D, green traces), and MICA-NKG2D (or ULBP3-NKG2D) complexes (red traces) in 1:2 binding molar ratios^4^,^5^. (**B**) Size exclusion chromatographic FPLC profiles and deconvolution of ULBP3-NKG2D mixtures at different molar ratios of ULBP3:NKG2D. ULBP3 was refolded by either our rapid method (“ULBP3 quick:NKG2D”; left panels) or traditional dialysis method (“ULBP3 dialysis:NKG2D”; right panels). In all panels, experimental mixture peaks are shown in black traces, simulated ULBP3-NKG2D peaks in red traces, extra NKG2D are shown as peaks in green, cumulative peaks of simulated ULBP3-NKG2D complex and extra NKG2D traces are shown in blue. Statistical evaluations of how close the experimental peaks to the simulated cumulative peaks are indicated by the R-square values (1.0 being a perfect fit). Detailed quantification of deconvoluted peak volumes and peak intensities are listed in Supplemental Table S3.

We also tested if purified receptor molecules are functionally active. We performed two sets of experiments. First, we used two cell surface ligands, MICA and ULBP3, in conjunction with their target receptor NKG2D as examples, because these receptor-ligand interactions have been well characterized^4^,^5^. Natural killer (NK) activating receptor NKG2D forms a stable complex with both MICA and ULBP3, both with a binding stoichiometry of 1:2 (ligand:receptor)^4^,^27^. The binding interactions were clearly visible by size exclusion chromatography as the elution profiles of either complex markedly shifted to shorter elution volumes (larger sizes of the complexes) when compared with the ligand or the receptor (Fig. 4A). Secondly, we tested if a ligand refolded by our rapid method binds to its receptor the same way as the same ligand refolded by traditional “dialysis” method. We used ULBP3 as an example. Using different ULBP3:NKG2D molar ratios (1:2, 1:3, 1:4, 1:6, and 1:8), each resulting size exclusion peak can be deconvoluted into two peaks: the ULBP3:NKG2D complex peak (molar ratio 1:2) and the peak for excess of NKG2D (deconvolution is necessary because the overlap between the ULBP3-NKG2D complex peaks and the peaks for the excess NKG2D). We observed that each set of deconvoluted peaks with ULBP3 made with the new rapid method can superimpose respective deconvoluted peaks with ULBP3 made with the dialysis method taking into account of experimental noises (Fig. 4 and Supplemental Table S3). We used peak intensity difference for the same amounts of ULBP3 refolded by the “rapid” and “dialysis” methods as the limit (10.1%) to tell if two deconvoluted peak intensities are superimposable or not. This applies to all ULBP3:NKG2D mixtures with different molar ratios (Fig. 4B and Supplemental Table S3). This binding data strongly suggest identical NKG2D-binding properties of ULBPs made from either method. These structural and functional characterizations demonstrated that refolded cell surface ligands and receptors from our new method were not only well-folded (except in the case of UL144), but also that they were functionally non-distinguishable with those refolded in typical dialysis approach in terms of their binding functions.

## Discussions

The method described in this report has several distinct advantages. First, our method efficiently couples modular on-column immobilized metal ion affinity purification and on-column protein refolding. We purposely avoid purification of inclusion bodies in their solid-state, which requires considerable amounts of time and reagents in inclusion body washing and centrifugation. In addition, the elution volume of refolded targets can be easily controlled to facilitate the concentration steps necessary for the follow-up purification step by size exclusion chromatography or a second affinity chromatography step. The entirety of the refolding and purification steps can be completed in one day, duration equivalent to that of the purification of soluble proteins. This is a significant improvement in efficiency compared to regular solid-state inclusion body purification followed by dilution and/or dialysis or approaches. In comparison with several cases of on-column refolding of proteins reported in the literature, our method provides an improvement in experimental efficiency and a general approach that works for a variety of membrane-associated receptors and ligands. Secondly, the format of on-column immobilization allows convenient follow-up processing of the refolded proteins. For example, if the refolded cell surface proteins are in the form of tagged or fusion proteins, it is facile to implement an enzymatic cleavage step. Thirdly, the current approach can be easily adapted to a high throughput format, for example, a 96-well plate or multi mini-column format. Such formats may have versatile applications in structural biology, chemical biology, and functional proteomics studies. Lastly, as demonstrated in the case of the UL37 exon 3 protein, we are able to refold this cell surface protein with our new method, but not with traditional “dialysis” or “dilution” approaches. Presumably, the on-column refolding format facilitates the refolding process, as reported in the case of EGFR domain refolding^24^.

We envision that our rapid receptor/ligand refolding method can incorporate other high throughput methods to develop powerful screening assays. A recent study reported DSF guided refolding as a novel method of protein production^13^. Using 96-well plates whose wells coated with Ni-NTA, our method can readily incorporate DSF analysis step *in situ*. The fluorescence-based assay can rapidly inform us about the optimal conditions for refolding cell surface proteins of interest. This refolding method can also couple with protein microarray and a means of analytical detection (such as fluorescence signals) to identify new binding ligands when applied to a collection of rapidly refolded cell surface receptors. Refolded receptors may be immobilized on carrier surfaces by affinity capture, noncovalent adsorption, or covalent binding^28^. For example, his_6_-tagged refolded receptors can be immobilized on Ni-NTA-coated glass slides. Receptor hits may be identified by label-dependent detection, such as Cyanine-3/5-labeled ligand of interest, or by label-free detection, such as surface plasmon resonance.

In summary, this study presents a rapid method for refolding and purifying host and microbial surface receptors and ligands. The method takes advantage of immobilized metal ion affinity purification and on-column solid-phase protein refolding. It skips lengthy inclusion body washing steps and dialysis steps associated with traditional refolding protocols. The robustness of our method is supported by the evidence that multiple classes of receptors with diverse structures may be refolded with comparable yields to those of classical dialysis or dilution methods. This method may be readily adapted into high throughput format, manufactured into a commercial kit, or adapted into automatic procedures. In the future, it will be particularly interesting to test our method with integral membrane proteins. We envision that this method will have significant applications in structural biology, functional proteomics, and chemical biology investigations.

## Methods

### Plasmids and Gene Synthesis

All 1expression vectors were either pET22b-based or pET15b-based plasmids. The plasmids generate either an amino-terminal or a carboxyl-terminal his_6_-tagged construct (Supplemental Table S1). Plasmids of MICA, γδ TCR, ULBP4, UL144, and UL146 were generously provided by Dr. Roland K. Strong (Fred Hutchinson Cancer Research Center, Seattle, WA). ULBP3 plasmid was a gift kindly provided by Dr. Peter D. Sun (NIH, Rockville, MD). Gene encoding HCMV UL37 × 3 was synthesized by GeneScript USA Inc. (Piscataway, NJ) and verified by DNA sequencing.

### Protein Expression, Refolding and Purification

NKG2D was refolded and purified according to the literature^4^. MICA, γδ TCR, ULBP3, ULBP4, and UL37 × 3 were expressed in *E. coli* BL21 (DE3) RIL CodonPlus strain (Stratagene, La Jolla, CA). UL144 and UL146 were expressed in *E. coli* strain BL21 (DE3). Bacteria were grown in 1L LB media containing 100 μg/ml ampicillin and/or 25 μg/ml chloramphenicol in 2.8 L Fernbach flasks. Cultures were grown at 37 °C until OD_600_ reaches 0.5–0.8 and were induced with 1 mM IPTG. UL37 × 3 culture was induced at 37 °C overnight. All the other proteins were induced at 37 °C for 4 hours. A 250-μl sample was taken immediately before and after induction for SDS-PAGE. Volume of the induced sample was determined based on its OD_600_. The sample was centrifuged at 15,000 rpm for 1 minute and the supernatant was discarded. Pellet was then re-suspended in 25 μL H_2_O, mixed with 25 uL 2xSDS sample buffer, denatured at 95 °C for 5 minutes. Freeze the sample at −20 °C until needed for running on SDS-PAGE. Cultures were collected by centrifugation at 9000 rpm for 30 minutes at 4 °C. The pellet was then re-suspended in 20–60 mLs of sonication lysis buffer (50 mM Tris pH 8.0, 150 mM NaCl and 0.5% Triton X-100) with freshly added 5 mM EDTA, and 5 mM DTT). The suspension was lysed using a Fisher sonication dismembrator (Model 300, Thermo Fisher Scientific Inc., Waltham, MA), and then incubated with 10 mM MgSO_4_ at room temperature for 20 minutes. The lysate was collected by centrifugation at 9000 rpm for 20 minutes at 4 °C and the pellet was saved for rapid refolding and traditional dialysis-based refolding.

For our rapid refolding method, the pellet was dissolved in dissolving buffer (10 mM Tris, 100 mM NaH_2_PO_4_, pH 8.0, and 8 M urea) for 2 hours at 4 °C while stirring. Dissolved inclusion bodies were cleared by centrifugation at 9000 rpm for 30 minutes at 4 °C. The supernatants were filtered through 110 μm Waterman filter membrane and were mixed with adequate volumes of Ni-NTA agarose resin (5 PRIME, Inc., Gaithersburg, MD) for 1 hour at 4 °C. The mixture was loaded onto a column. The resin was washed with 40 times volume of dissolving buffer and 40 times volume of washing buffer (10 mM Tris, 100 mM NaH_2_PO_4_, pH 8.0, 8 M urea, and 20 mM imidazole). Refolding was performed on column with 40 times volume of refolding buffers containing 3 M urea and 1.5 M urea respectively. The resin was washed with sufficient volume of phosphate buffer saline (1xPBS) and eluted with elute buffer (1xPBS pH 8.0, and 200 mM imidazole). The eluted proteins were concentrated and injected into HiLoad 16/60 Superdex 75 gel filtration column attached with ÄktaFPLC system (GE Healthcare Bio-Sciences, Pittsburgh, PA). The fractions were collected and used for protein quantification and for quality check by running SDS-PAGE.

For traditional dialysis-based refolding of ULBP3 and UL146, both were expressed and lysed as described above. The lysates were collected by centrifugation at 9000 rpm for 20 minutes at 4 °C. The pellets were washed three times with washing buffer A (50 mM Tris pH 8.0, 0.1 M NaCl, 1 mM DTT, 0.5% Triton X-100) and once with buffer B (50 mM Tris pH 8.0, 2 M NaCl, 1 mM DTT, and 1 M urea). The washing/resuspending-centrifugating steps were repeated multiple times (typically 8–14 times) until the inclusion bodies were 90–95% pure estimated by SDS-PAGE. The pellets were dissolved in dissolving buffer (10 mM Tris, 100 mM NaH_2_PO_4_, pH 8.0, and 8 M urea) overnight at 4 °C while stirring. Dissolved inclusion bodies were cleared by centrifugation at 9000 rpm for 30 minutes at 4 °C. The supernatants were filtered through 110 μm Waterman filter membrane and were mixed with adequate volumes of N-NTA agarose resin for 1 hour at 4 °C. The mixtures were loaded onto a column. The resins were washed with 15 times volume of dissolving buffer and 15 times volume of washing buffer (10 mM Tris, 100 mM NaH_2_PO_4_, pH 8.0, 8 M urea, and 20 mM imidazole). The inclusion bodies were eluted with elusion buffer (10 mM Tris, 100 mM NaH_2_PO_4_, pH 8.0, 8 M urea, and 200 mM imidazole). The eluted, purified inclusion bodies were diluted to 0.3 mg/ml and loaded into a dialysis tubing with suitable molecular weight cut-off for refolding. Refolding and purification processes were followed by a regular dialysis-based refolding protocol as described previously^4^,^9^.

### Protein Structure Modeling

Structure models of MICA, γδ TCR, and ULBP3 were generated using PyMol according to PDB code 1HYR, 3OMZ, and 1KCG respectively^4^,^5^,^9^. Structure models of ULBP4, UL37 × 3, and UL146 were generated using Phyre2 protein fold recognition server^29^. Structure model of UL144 was generated using I-TASSER server^30^.

### Circular Dichroism Spectroscopy

The spectra were acquired using a JASCO J-815 circular dichroism spectrometer over the wavelength range from 190 to 250 nm. All measurements were performed at 20 °C. The spectra were acquired using 5-20 μM protein concentrations in 10 mM potassium phosphate buffer (pH 7.0) in a 1 mm path-length quarts cell (Starna Cells, Inc., Atascadero, CA). Buffer spectra were subtracted from the raw data. The ellipticity was calculated following reference formulas: Ellipticity, [θ], in deg cm^2^ dmol^−1^ = millidegrees/(pathlength in millimeters × the molar concentration of protein × the number of residues) following literature^31^.

### Differential Scanning Fluorimetry

Optimal concentrations of 20 μM of protein and 3 μM Sypro Orange dye (ThermoFisher, Grand Island, NY) were used in the protein samples. The samples were added to a 96-well plate and the spectra were acquired using a Bio-Rad real-time PCR detection system (Bio-Rad, Hercules, CA). The melting temperature range was set from 4–95 °C with increment of 6 °C per minute. The fluorescence was monitored using standard excitation/emission wavelengths, and the protein Tm was determined using the manufacturer’s software. Thermo-unfolding profile of a sample of buffer only was also acquired and was used as background control to be subtracted in data analyses. The RFU values from different data sets were normalized to a common scale of 0–1 as described in the literature^32^.

### Receptor Binding Assay

For MICA and NKG2D binding assay, 0.8 nmol of MICA and 1.6 nmol of NKG2D (molar ratio 1:2) were mixed together and injected into Superdex 75 10/300 gel filtration column attached with ÄktaFPLC chromatographic system. Elution profile of the complex was compared with those of MICA alone or NKG2D only. For ULBP3 and NKG2D binding assay, different amounts of ULBP3 (2.5 nmoles, 1.667 nmols, 1.25 nmols, 0.9375 nmoles, and 0.625 nmoles) and 5 nmol of NKG2D were mixed to give ULBP3:NKG2D molar ratios of 1:2, 1:3, 1:4, 1:6, and 1:8. The mixtures were analyzed using similar procedures described for the MICA-NKG2D complex. The mixture peaks were deconvoluted using programs from OriginPro (OriginLab Corporation, Northampton, MA) and the deconvulated peak intensities and peak elution volumes were compared.

## Additional Information

**How to cite this article**: Zhai, L. *et al.* A Rapid Method for Refolding Cell Surface Receptors and Ligands. *Sci. Rep.* **6**, 26482; doi: 10.1038/srep26482 (2016).

## Supplementary Material

Supplementary Information

## Figures and Tables

**Table 1:** Comparison of yields of refolded target proteins with this rapid method and traditional “dialysis” method.

Protein	Yield		
	This method	Dialysis	Literature
MIC-A	1.0 mg/L	1–2 mg/L	1.1 mg/L^a^
γδ TCR	0.7 mg/L	1–3 mg/L	
ULBP3	1.5 mg/L	1.6–2.2 mg/L	Not reported^b^
ULBP4	0.8 mg/L	0.5–1.0 mg/L	
HCMV UL37 × 3	0.6 mg/L	Not successful^c^	
HCMV UL144	0.8 mg/L	1–2 mg/L	
HCMV UL146	2.6 mg/L	5 mg/L	

^a^Value obtained from literature^33^.

^b^ULBP3 was refolded using dilution approach, but value was not reported^5^.

^c^No UL37 × 3 was successfully refolded using dialysis approach.
