# Supplementary material for: A Rapid Method for Refolding Cell Surface Receptors and Ligands
Source: Sci Rep. 2016 May 24;6:26482. doi: 10.1038/srep26482 (PMC4877712; doi:10.1038/srep26482)
Supplement: Supplementary Information [file srep26482-s1.pdf]

A Rapid Method for Refolding Cell Surface Receptors and Ligands

Lu Zhai, Ling Wu, Feng Li, Robert S. Burnham, Juan C. Pizarro, and Bin Xu

Supplementary Information

Supplementary Table 1. Amino acid sequences of the targeted immune- and viral surface receptors and ligands. Protein sequences are in standard single letter code format. Each construct contains a his_6-tag either in the amino-terminus or carboxyl-terminus of the protein.

Protein	Sequence
**MICA**	MEPHSLRYNLTVLSWDGSVQSGFLTEVHLDGQPFLRCDRQKCRAKPQGQWAEDVLGNKTWDRETRDLTG<br>NGKDLRMTLAHIKDQKEGLHSLQEIRVCEIHEDNSTRSSQHFYYDGELFLSQNLETKEWTMPQSSRAQT<br>LAMNVRNFLKEDAMKTKTHYHAMHADCLQELRRYLKSGVVLRRTVPPMVNVTRSEASEGNITVTCRASG<br>FYPWNITLSWRQDGVSLSHDTQQWGDVLPDGNGTYQTWVATRICQGEEQRFTCYMEHSGNHSTHPVPSH<br>HHHHH
**γδ TCR**	MSSNLEGRTKSVIRQTGSSAEITCDLAEGSTGYIHWYLHQEGKAPQRLLYYDSYTSSVVLESGISPGKY<br>DTYGSTRKNLRMILRNLIENDSGVYYCATWDQNYYKKLFGSGTSLVVTDKQGSADDAKKDAAKKDGAQK<br>VTQAQSSVSMPVRKAVTLNCLYETSWWSYYIFWYKRLPSKEMIFLIRQGSDEQNAKSGRYSVNFKKAAK<br>SVALTISALQLEDSAKYFCALGESLTRADKLIFGKGTRVTVEPRSQHHHHHH
**ULBP3[a]**	MFDWSGTGRADAHSLWYNFTIIHLPRHGQQWCEVQSQVDQKNFLSYDCGSDKVLSMGHLEEQLYATDAW<br>GKQLEMLREVGQRLRLELADTELEDFTPSGPLTLQVRMSCECEADGYIRGSWQFSFDGRKFLLFDSNNR<br>KWTVVHAGARRMKEKWEKDSGLTTFFKMVSMRDCKSWLRDFLMHRKKRLEPTAPPTMAPGLAQPLEHHH<br>HHH
**ULBP4**	MHSLCFNFTIKSLSRPGQPWCEAQVFLNKNLFLQYNSDNNMVKPLGLLGKKVYATSTWGELTQTLGEVG<br>RDLRMLLCDIKPQIKTSDPSTLQVEMFCQREAERCTGASWQFATNGEKSLLFDAMNMTWTVINHEASKI<br>KETWKKDRGLEKYFRKLSKGDCDHWLREFLGHWEAMPEPTHHHHHH
**UL37X3**	MHGVSVRCTYHGTDVNRTSNTTSMNCHLNCTRNHTQIYNGPCLGTEARLPLNVTFNQSRRKWHSVMLKF<br>GFQYHLEGWFPLRVLNESREINVTEVHGEVACFRNDTNVTVGQLTLNFTGHSYVLRAIAHTSPFESYVR<br>WEETNVTDNATSSENTTTVMSTLTKYAESDYIFLQDMCPRFLKRTVKLTRNKTKLVPRGSHHHHHH
**UL144**	MHHHHHHGSLVPRGSKVCQHNEVQLGNECCPPCGLGQRVTKVCTERTSVTCTPCPNGTYVSGLYNCTDC<br>TQCNVTQVMIRNCTSTNNTVCAPKNHTYFSTPGVQHHKQRQQNHTAHITVKQGKSGRH
**UL146**	MRLIFGALIISLTYMYYYEVHGTELRCKCLDGKKLPPKTIMLGNFWFHRESGGPRCNNNEYFLYLGGGK<br>KHGPGVCLSPHHPFSKWLDKRNDNRWYNVNVTRQPERGPGKITVTLVGLKEHHHHHH

[a] See reference for details[5].

Supplementary Table 2. Comparison of yields of refolded ULBP3 and UL146 using this rapid method and traditional "dialysis" method.

Step	ULBP3		UL146	
	**This method**	**Dialysis**	**This method**	**Dialysis**
**Expressed**	90 mg	90 mg	110 mg	110 mg
**Crude IBs**		75 mg		N/A
**Cleaned IBs**		24 mg		N/A
**Purified from Ni-NTA**		21 mg		75 mg
**Refolded**	2.2 mg	3.0 mg	3.8 mg	7.2 mg
**Final yields**	1.5 mg	2.2 mg	2.62 mg	5 mg

Supplementary Table 3. Quantification and comparison of deconvoluted peaks in ULBP3-NKG2D binding with ULBP3 refolded by this rapid method or traditional "dialysis" method.

Peak Name	Peak Elute Volume (ml)		Elute Volume Difference	Peak Intensity (mAU)		Peak Intensity Difference
**Control**						
NKG2D	12.88			14.46		
**Control**	**Rapid**	**Dialysis**		**Rapid**	**Dialysis**	
ULBP3	12.41	12.40	0.08%	14.34	15.95	10.1%
**ULBP3:NKG2D (1:8)**	**Rapid**	**Dialysis**		**Rapid**	**Dialysis**	
Simulated ULBP3-NKG2D complex	12.41	12.44	0.24%	3.36	3.08	8.33%
Simulated NKG2D	12.87	12.87	0	11.62	12.34	5.83%
**ULBP3:NKG2D (1:6)**	**Rapid**	**Dialysis**		**Rapid**	**Dialysis**	
Simulated ULBP3-NKG2D complex	12.41	12.39	0.16%	5.28	5.65	6.55%
Simulated NKG2D	12.87	12.89	0.16%	10.05	10.92	7.97%
**ULBP3:NKG2D (1:4)**	**Rapid**	**Dialysis**		**Rapid**	**Dialysis**	
Simulated ULBP3-NKG2D complex	12.40	12.36	0.32%	8.38	8.76	4.34%
Simulated NKG2D	12.86	12.89	0.23%	7.54	7.72	2.33%
**ULBP3:NKG2D (1:3)**	**Rapid**	**Dialysis**		**Rapid**	**Dialysis**	
Simulated ULBP3-NKG2D complex	12.29	12.30	0.08%	11.37	11.20	1.50%
Simulated NKG2D	12.87	12.91	0.31%	6.68	6.50	2.69%
**ULBP3:NKG2D (1:2)**	**Rapid**	**Dialysis**		**Rapid**	**Dialysis**	
ULBP3-NKG2D complex	12.22	12.27	0.41%	13.50	14.94	9.63%

Supplementary Figure 1. Schematic flow-charts for three different refolding methods with estimated time required for completing each step.

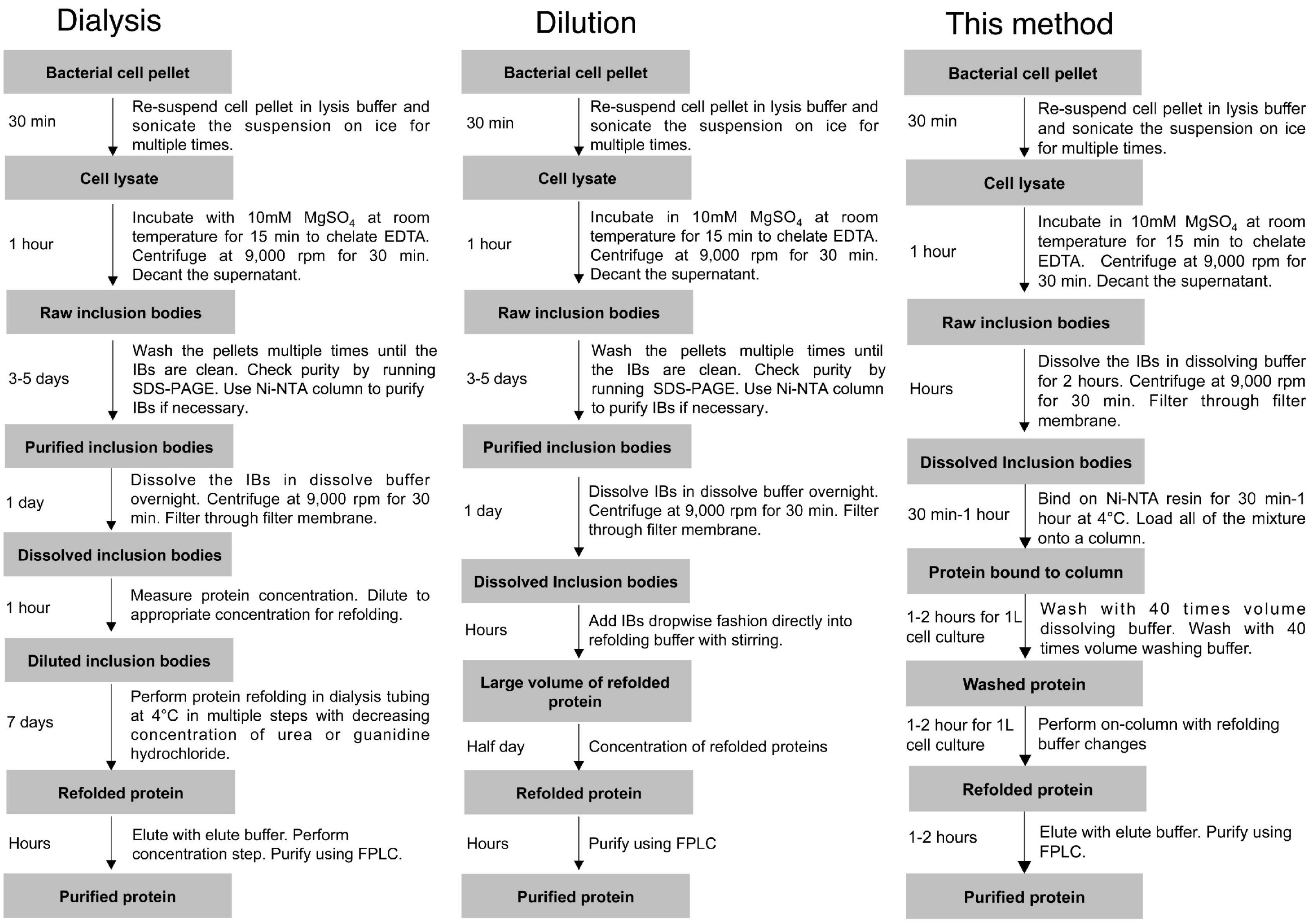
Dialysis
Bacterial cell pellet
30 min
Re-suspend cell pellet in lysis buffer and sonicate the suspension on ice for multiple times.
Cell lysate
1 hour
Incubate with 10mM $MgSO_4$ at room temperature for 15 min to chelate EDTA. Centrifuge at 9,000 rpm for 30 min. Decant the supernatant.
Raw inclusion bodies
3-5 days
Wash the pellets multiple times until the IBs are clean. Check purity by running SDS-PAGE. Use Ni-NTA column to purify IBs if necessary.
Purified inclusion bodies
1 day
Dissolve the IBs in dissolve buffer overnight. Centrifuge at 9,000 rpm for 30 min. Filter through filter membrane.
Dissolved inclusion bodies
1 hour
Measure protein concentration. Dilute to appropriate concentration for refolding.
Diluted inclusion bodies
7 days
Perform protein refolding in dialysis tubing at 4°C in multiple steps with decreasing concentration of urea or guanidine hydrochloride.
Refolded protein
Hours
Elute with elute buffer. Perform concentration step. Purify using FPLC.
Purified protein
Dilution
Bacterial cell pellet
30 min
Re-suspend cell pellet in lysis buffer and sonicate the suspension on ice for multiple times.
Cell lysate
1 hour
Incubate in 10mM $MgSO_4$ at room temperature for 15 min to chelate EDTA. Centrifuge at 9,000 rpm for 30 min. Decant the supernatant.
Raw inclusion bodies
3-5 days
Wash the pellets multiple times until the IBs are clean. Check purity by running SDS-PAGE. Use Ni-NTA column to purify IBs if necessary.
Purified inclusion bodies
1 day
Dissolve IBs in dissolve buffer overnight. Centrifuge at 9,000 rpm for 30 min. Filter through filter membrane.
Dissolved Inclusion bodies
Hours
Add IBs dropwise fashion directly into refolding buffer with stirring.
Large volume of refolded protein
Half day
Concentration of refolded proteins
Refolded protein
Hours
Purify using FPLC
Purified protein
This method
Bacterial cell pellet
30 min
Re-suspend cell pellet in lysis buffer and sonicate the suspension on ice for multiple times.
Cell lysate
1 hour
Incubate in 10mM $MgSO_4$ at room temperature for 15 min to chelate EDTA. Centrifuge at 9,000 rpm for 30 min. Decant the supernatant.
Raw inclusion bodies
Hours
Dissolve the IBs in dissolving buffer for 2 hours. Centrifuge at 9,000 rpm for 30 min. Filter through filter membrane.
Dissolved Inclusion bodies
30 min-1 hour
Bind on Ni-NTA resin for 30 min-1 hour at 4°C. Load all of the mixture onto a column.
Protein bound to column
1-2 hours for 1L cell culture
Wash with 40 times volume dissolving buffer. Wash with 40 times volume washing buffer.
Washed protein
1-2 hour for 1L cell culture
Perform on-column with refolding buffer changes
Refolded protein
1-2 hours
Elute with elute buffer. Purify using FPLC.
Purified protein

Supplementary Figure 2. Comparison of size exclusion chromatographic FPLC profiles of ULBP3 refolding optimized with different reducing/oxidizing pairs of reagents (left panels) and with different molar ratios of GSH/GSSG (right panel).

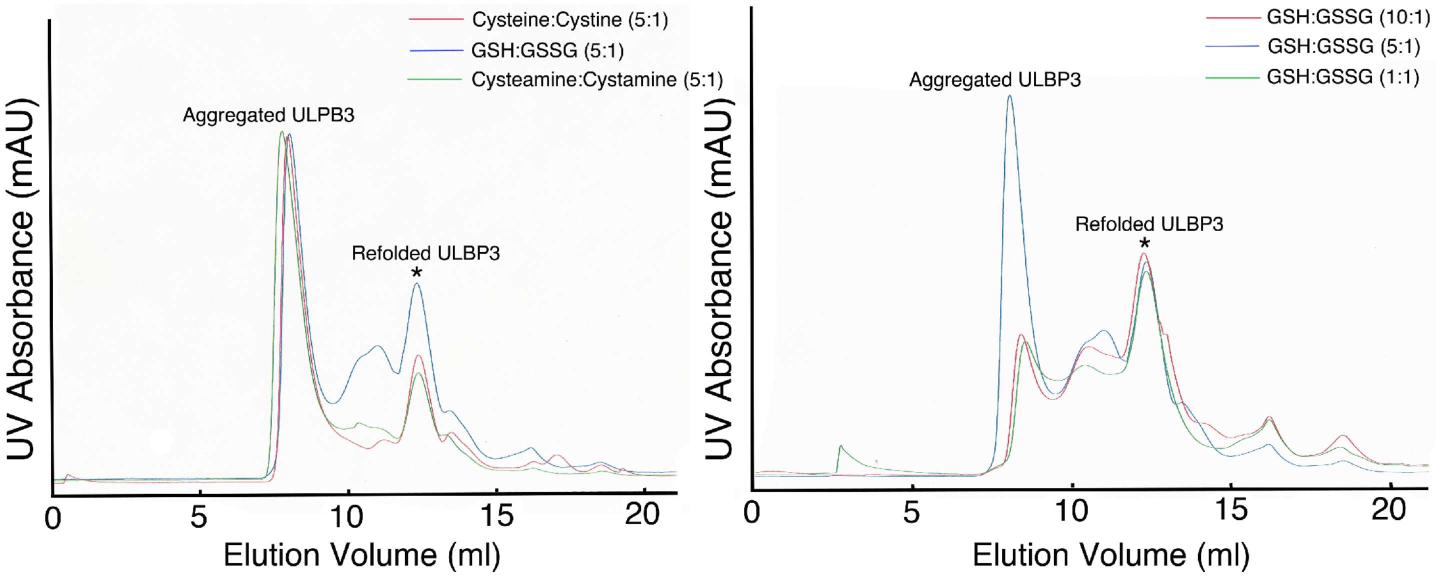

Cysteine:Cystine (5:1)
GSH:GSSG (5:1)
Cysteamine:Cystamine (5:1)
Aggregated ULPB3
Refolded ULBP3
*
UV Absorbance (mAU)
0
5
10
15
20
Elution Volume (ml)
GSH:GSSG (10:1)
GSH:GSSG (5:1)
GSH:GSSG (1:1)
Aggregated ULBP3
Refolded ULBP3
*
UV Absorbance (mAU)
0
5
10
15
20
Elution Volume (ml)
